# The role of pulse indicator continuous cardiac output (PiCCO) and critical care ultrasound in volume status assessment during fluid resuscitation for and prognosis of septic shock patients

**DOI:** 10.12669/pjms.39.1.5603

**Published:** 2023

**Authors:** Yuxin Luo, Sidong Zhan, Lijuan Zhu, Meilan Xiong, Guiyan Liu, Cailing Wang

**Affiliations:** 1 Yuxin Luo, Emergency Intensive Care Unit, Huizhou Central People’s Hospital, Huizhou 516001, Guangdong, China; 2 Sidong Zhan, Emergency Intensive Care Unit, Huizhou Central People’s Hospital, Huizhou 516001, Guangdong, China; 3 Lijuan Zhu, Emergency Intensive Care Unit, Huizhou Central People’s Hospital, Huizhou 516001, Guangdong, China; 4 Meilan Xiong, Emergency Intensive Care Unit, Huizhou Central People’s Hospital, Huizhou 516001, Guangdong, China; 5 Guiyan Liu, Emergency Intensive Care Unit, Huizhou Central People’s Hospital, Huizhou 516001, Guangdong, China; 6 Cailing Wang, Emergency Intensive Care Unit, Huizhou Central People’s Hospital, Huizhou 516001, Guangdong, China

**Keywords:** Septic shock, Pulse index continuous cardiac output (PiCCO), Critical care ultrasound, Volume status assessment, Prognosis

## Abstract

**Objectives:**

To investigate whether pulse index continuous cardiac output (PiCCO) and critical care ultrasound are highly consistent in volume status assessment during fluid resuscitation for septic shock patients and analyze their influence on the prognosis of septic shock.

**Methods:**

Eighty septic shock patients treated by Huizhou Central People’s Hospital during December 2018 and December 2020 were included and divided into a study group and a control group by the presence of volume responsiveness, with each group having 40 patients. The control group was subject to PiCCO-guided fluid resuscitation therapy, while the study group was given fluid resuscitation therapy guided by critical care ultrasound. Cardiac output, cardiac function, and catheter-related infection (CRI) were documented for intergroup comparison to confirm whether these two techniques were consistent with each other regarding their effects on resuscitation for and prognosis of septic shock patients.

**Results:**

Mechanical ventilation duration (MVD) and intensive care unit (ICU) length of stay (LoS) were significantly shorter in the study group when compared with the control group, and the differences were statistically significant (p<0.05, respectively). In terms of blood pressure parameters, the two groups did not differ greatly in diastolic blood pressure (DBP), mean arterial pressure (MAP), systolic blood pressure (SBP), and central venous pressure (CVP) before resuscitation (p>0.05, respectively); at 6h(six hour) after resuscitation, DBP, MAP, SBP, and CVP were substantially increased in both groups as compared with the pre-resuscitation levels (all p<0.05), but the differences between the two groups lacked statistical significance (all p>0.05). Comparing urine volume and degrees of positive fluid balance at 6 h and 12 h after resuscitation, drastic increases in urine volume and positive fluid balance were observed in both groups at 12 h as compared with at 6 h (all p<0.05); nevertheless, the two groups showed no statistically significant difference in urine volume and positive fluid balance at 6 h or 12 h (p>0.05, respectively). With regards to prognosis, there was no statistically significant difference between the two groups in the number of cases of continuous renal replacement therapy (CRRT), dosage of vasoactive agents and 28-d mortality rate (all p>0.05). However, the incidence of CRI was markedly lower in the study group (0/40) as compared with the control group (5/40), and the difference was statistically significant (p<0.05).

**Conclusions:**

Both PiCCO and critical care ultrasound can help achieve favorable outcomes from resuscitation for septic shock patients. Compared with PiCCO, critical care ultrasound monitoring appears to be more effective in preventing CRI and reducing MVD and ICU LoS, thereby easing patients’ medical burden.

## INTRODUCTION

Septic shock is a form of distributive shock induced by low blood pressure and insufficient perfusion attributed to sepsis contributed by cells undergoing hypoxia and ischemia associated with diverse causative factors. The condition is characterized by distributive hypovolemia, which indicates a need for sufficient fluid resuscitation.^1^,^2^ However, fluid resuscitation with an inadequate volume may fail in relieving shock because of insufficient perfusion, while fluid resuscitation with an excessive volume is highly likely to induce pulmonary edema and other complications and adversely affect the prognosis because the body is overloaded.^3^,^4^ Therefore, accurate hemodynamic monitoring is essential for resuscitation therapy for septic shock patients. Pulse index continuous cardiac output (PiCCO) is a clinical method commonly used to monitor fluid resuscitation. Despite its validated benefits, PiCCO is found to somewhat affect prognosis as an invasive monitoring method in the clinical setting.^5^ As medical technology advances, critical care ultrasound has gained wider clinical application as a point-of-care ultrasound technique to treat patients faster in a non-invasive, radiationless and low-cost way.^6^ To further explore the significance of critical care ultrasound in resuscitation for septic shock patients and analyze its effect on the prognosis of these patients, a controlled study was conducted using PiCCO and critical care ultrasound for patient monitoring.

## METHODS

A total of 80 septic shock patients who were admitted and treated by Huizhou Central People’s Hospital during December 2018 and December 2020 were included in this study and assigned to a study group (n =40) and a control group (n =40) according to the presence of volume responsiveness (one was rendered volume responsive when the difference of stroke volume index (SVI) before and after fluid infusion was over 10%-15%). The study group consisted of 26 male and 14 female patients at the age between 33 and 76 years, with the mean age of (57.31 ±3.92) years. The control group had 28 male and 12 female patients who were 36 to 78 years old, and the mean age was (57.33 ±3.96) years. The two groups did not differ greatly in baseline characteristics (p>0.05), which indicated a high degree of comparability.

### Inclusion criteria:

- confirmed to have septic shock;
- at the age of 18 to 80;
- participation with informed consent.

### Exclusion criteria:

- having contraindications for PiCCO or critical care ultrasound monitoring;
- present with severe arrhythmia;
- accompanied with serious organ dysfunction;
- diagnosed with conscious disturbance;
- early withdrawal.

### Ethical Approval:

The study was approved by the Institutional Ethics Committee of Huizhou Central People’s Hospital (No.: kyll2021064; Date: April 22, 2021), and written informed consent was obtained from all participants.

The control group received PiCCO-guided fluid resuscitation therapy: Each patient lay in a prostrate position as instructed, and a venous duct (ARROW, REF CS-24301-E) was inserted into the subclavian vein; following that, the PiCCO catheter (PULSION Medical Systems SE, PV2015L20-A) was retained in the femoral artery and connected to the PiCCO monitor (Philips InteliVue MP60, M1012A) to guide fluid resuscitation based on the monitoring results.

The study group underwent fluid resuscitation therapy guided by critical care ultrasound (PHILIPS Saronno ITALY, MCMDO2AA). To gain a clearer picture of the systolic function, ultrasonography was performed by scanning the lower left of the chest with a cardiac probe (frequency: 3 Mhz) to obtain the long axis view and observe the chambers of the heart. Subsequently, the ultrasound system was switched to the M mode, and the sample line was placed at the mitral chordae tendineae to monitor the end-systolic and end-diastole diameters of the left ventricle. To acquire data from the apical four-chamber view, the cardiac probe was placed at the cardiac apex. Then, end-systolic and end-diastolic left ventricular volume, cardiac output, and left ventricular ejection fraction were generated automatically by the ultrasound system.

### Outcome measures:

(1) Clinical indicators: mechanical ventilation duration (MVD) and ICU length of stay (LoS); (2) diastolic blood pressure (DBP), mean arterial pressure (MAP), systolic blood pressure (SBP), and central venous pressure (CVP): time of data collection: before resuscitation and at 6 h after resuscitation; parameters: DBP, MAP, SBP, and CVP; (3) urine volume and positive fluid balance: time of data collection: at 6 h and 12 h after resuscitation; parameters: urine volume and positive fluid balance; (4) prognosis: comparison of 28-d mortality rate, dosage of vasoactive agents, number of cases of continuous renal replacement therapy (CRRT) and catheter-related infection (CRI) between the two groups.

### Statistical Analysis:

The software SPSS22.0 was used for data processing. Enumeration data were expressed by “n(%)”, and intergroup comparison of male-to-female ratio, number of CRRT cases, 28-d mortality rate, and number of CRI cases were examined by the χ2 test. Measurement data were represented by (*χ̄*±*S*), and intergroup comparison of age, MVD, ICU LoS, DBP, MAP, SBP, CVP, urine volume, positive fluid balance, and dosage of vasoactive agents were examined by the t-test. P<0.05 indicates a difference of statistical significance.

## RESULTS

MVD and ICU LoS were significantly shorter in the study group when compared with the control group (p<0.05). Table-I. Before resuscitation, there was no statistically significant difference between the two groups in DBP, MAP, SBP, or CVP (p>0.05, respectively); at 6 h after resuscitation, DBP, MAP, SBP, and CVP in both groups were increased significantly as compared with the pre-resuscitation levels (all p<0.05), but the differences between the two groups had no statistical significance (p>0.05, respectively). Table-II. At 12 h after resuscitation, an increased urine volume and a higher degree of positive fluid balance were observed in both groups when compared with those at 6 h after resuscitation, and the differences were statistically significant (p<0.05, respectively); however, there was no statistically significant difference between the two groups in the level of urine volume or degree of positive fluid balance at 6 h or 12 h after resuscitation (p>0.05, respectively). Table-III. The two groups did not differ in the number of CRRT cases, dosage of vasoactive agents or 28-d mortality rate (p>0.05, respectively); the incidence of CRI in the study group (0/40) was significantly lower than in the control group (5/40) (p<0.05). Table-IV.

**Table-I:** Comparison of clinical indicators (*χ̅*±*S*)

Group	n	MVD (d)	ICU LoS (d)
Study group	40	7.55±2.18	14.21±2.48
Control group	40	9.96±3.21	18.42±2.79
*t*-value	-	3.928	7.133
*p*-value	-	<0.001	<0.001

**Table-II:** Comparison of blood pressure parameters before and at 6 h after resuscitation (*χ̅*±*S*).

Group	n	DBP (mmHg)			

		Pre-resuscitation	At 6 h post-resuscitation	t-value	p-value
Study group	40	52.57±9.16	64.51±12.55	4.860	<0.001
Control group	40	53.06±9.22	65.13±12.62	4.884	<0.001
*t-value*	-	0.238	0.22	-	-
*P-value*	-	0.812	0.826	-	-

*Group*	*n*	*MAP (mmHg)*			

		*Pre-resuscitation*	*At 6 h post-resuscitation*	*t-value*	*P-value*

Study group	40	64.08±8.84	78.52±9.64	6.982	<0.001
Control group	40	64.21±9.13	79.15±9.86	7.032	<0.001
*t-value*	-	0.065	0.289	-	-
*P-value*	-	0.949	0.773	-	-

*Group*	*n*	*SBP (mmHg)*			

		*Pre-resuscitation*	*At 6 h post-resuscitation*	*t-value*	*P-value*

Study group	40	89.32±13.54	122.28±15.64	10.077	<0.001
Control group	40	89.18±13.46	122.31±15.68	10.140	<0.001
*t-value*	-	0.046	0.009	-	-
*P-value*	-	0.963	0.993	-	-

*Group*	*n*	*CVP (mmHg)*			

		*Pre-resuscitation*	*At 6 h post-resuscitation*	*t-value*	*P-value*

Study group	40	7.16±2.03	13.17±2.74	11.147	<0.001
Control group	40	7.21±2.08	13.22±2.88	10.699	<0.001
*t-value*	-	0.109	0.08	-	-
*p-value*	-	0.914	0.937	-	-

**Table-III:** Comparison of urine volume and positive fluid balance at 6 h and 12 h after resuscitation (χ̅±s).

Group	n	Urine volume (mL)			

		At 6 h post-resuscitation	At 12 h post-resuscitation	t-value	p-value
Study group	40	255.49±28.64	618.46±162.27	13.932	<0.001
Control group	40	249.42±26.61	609.48±160.21	10.802	<0.001
*t-value*	-	0.982	0.249	-	-
*P-value*	-	0.329	0.804	-	-

Group	*n*	Positive fluid balance (mL)			

		At 6 h post-resuscitation	At 12 h post-resuscitation	*t-value*	*P-value*

Study group	40	1052.16±148.23	1532.27±198.54	12.255	<0.001
Control group	40	1065.18±152.26	1540.29±201.58	16.160	<0.001
*t-value*	-	0.388	0.179	-	-
*P-value*	-	0.699	0.858	-	-

**Table-IV:** Comparison of prognosis [n (%)].

Group	n	CRRT case(s)	Dosage of vasoactive agents (mL/h)	28-d mortality rate (%)	CRI case(s)
Study group	40	3(7.5)	4.16±1.49	9(22.5)	0(0.00)
Control group	40	5(12.5)	4.57±1.52	11(27.5)	5(12.5)
t/χ2-value	-	0.556	1.218	0.267	5.333
p-value	-	0.456	0.227	0.606	0.021

## DISCUSSION

Septic shock is also known as infectious shock because it can give rise to metabolic disorders, cellular ischemia and hypoxia, and dysfunction, resulting in an extremely high mortality rate.^7^,^8^ Septic shock is also an important lethal factor among ICU patients.^9^ Fluid resuscitation is currently a commonly used clinical therapy for septic shock patients. However, excessive fluid resuscitation entails a risk of pulmonary edema that affects the patient’s prognosis; on the other hand, insufficient resuscitation cannot improve the condition. Therefore, it is essential to achieve accurate fluid management during resuscitation.^10^,^11^

PiCCO is a clinically common tool to transform pressure monitoring into volume monitoring and support data generation without massive invasion.^12^ This study showed that at 6 h after resuscitation, DBP, MAP, SBP, and CVP were substantially increased in both groups when compared with the pre-resuscitation levels; the two groups did not differ in DBP, MAP, SBP, or CVP at 6 h after resuscitation. In addition, comparing the levels of urine volume and the degrees of positive fluid balance at 6 h and 12 h after resuscitation, significant increases were observed at 12 h as compared with at 6 h after resuscitation; the two groups had no statistically significant difference in urine volume or positive fluid balance between the two groups at 6 h or 12 h after resuscitation.^13^ The study results suggested that PiCCO and critical care ultrasound could facilitate fluid resuscitation, regulate urine volume and modulate positive fluid balance, consistent with the results in Yu et al. However, clinical evidence shows that as an invasive tool, PiCCO has an adverse impact on patient’s prognosis.^14^

Critical care ultrasound is an efficient, non-invasive, radiationless, and low-cost technique that has gained increasingly extensive use in critical care medicine.^15^,^16^ The study results showed that the study group had significantly shorter MVD and ICU LoS compared with the control group. Moreover, the incidence of CRI in the study group (0/40) was significantly lower than in the control group (5/40). This might be explained by the benefits of critical care ultrasound as an efficient, noninvasive, radiation less and low-cost technique that enables repetitive inspections and comprehensive assessment. On this basis, clinicians can make necessary adjustments to treatment regimens and relieve patients’ conditions. Compared with traditional ultrasonography, critical care ultrasound supports centralized monitoring of lesions in an efficient manner, which dramatically reduces the delay time during the process and thus is suitable for monitoring septic shock with unstable hemodynamic conditions.^17^,^19^ In the meantime, this study also suggested that the two groups did not differ in the number of CRRT cases, dosage of vasoactive agents or 28-d mortality, which demonstrated that although both techniques could guide resuscitation, they provided no guarantee for a death risk-free state.

## CONCLUSION

Both PiCCO and critical care ultrasound can help achieve favorable outcomes from resuscitation for septic shock patients. Compared with PiCCO, critical care ultrasound monitoring appears to be more effective in preventing CRI and reducing MVD and ICU LoS, thereby easing patients’ medical burden.

### Authors’ Contributions:

**YL &** **SZ:** Designed this study, prepared this manuscript, are responsible and accountable for the accuracy or integrity of the work.

**LZ &** **MX:** Collected and analyzed clinical data.

**GL &** **CW:** Data analysis, significantly revised this manuscript.
